# Impact of changes in regulatory framework on approval of medicines for rare diseases and applicability to market access policies

**DOI:** 10.3389/fmed.2025.1474087

**Published:** 2025-04-02

**Authors:** Fran Brown, Maximilian Vargas, Sanja Stanisic, Geoff Fatzinger, Oxana Iliach

**Affiliations:** ^1^Certara, Radnor, PA, United States; ^2^Certara, Toronto, ON, Canada

**Keywords:** rare disease, orphan drug, market access, regulatory policy, approval, marketing

## Abstract

The introduction of the Orphan Drug Act in the USA in 1983, followed by adoption of the Orphan Drug Regulation No 141/2000 in the EU in 2000, led to a change in landscape of drug development for rare diseases. The introduction of regulations, guidance documents and incentives aimed at increasing the availability of new medicines for rare diseases resulted in an increase in approvals of 3 and 11-fold for branded products and generic medicines, respectively, in the decade 2013–2023 compared to 1990–2000. This effort was successful due to the collaboration of Regulatory Authorities, industry, patient groups and other stakeholders keen to leverage an integrated evidence approach using non-traditional approaches. While the regulatory approval landscape moved toward integration, the effective access to those medicines over the same period was globally fragmented with pricing and access determined at a local level. There is growing recognition of the importance of addressing the needs of rare disease patients and a concerted effort to balance innovation with affordability and access.

## Introduction

The development landscape for new medicines for rare diseases has undergone significant changes over the last two decades. At the beginning of the 21st century, many countries had limited or non-specific legislation for rare diseases. Orphan drug policies were just starting to gain traction, with only a few countries implementing dedicated programs. The Orphan Drug Act in the United States (1983) had already set a precedent for rare disease drug development by offering incentives like tax credits, grant funding, and market exclusivity (1). European countries started to adopt some policies, though they varied in scope and implementation. Despite these attempts to address some of the barriers which prevented the development of drugs for rare diseases, incentives for pharmaceutical companies were insufficient to offset the high costs and risks associated with these medicines, until implementation of Orphan Drug Regulation No 141/2000 in 2000 (4). In this paper we attempt to quantitate the impact of the introduction of those regulations and associated guidance documents on the availability of new medicines for rare diseases and assess the drivers behind the outcomes we observe. We recognize and acknowledge that in addition to the guidances there were multiple additional incentives introduced to facilitate development and approval of drugs for rare diseases. However, we focus our review on the guidances to establish any potential correlation with the access to these therapies. Moreover, we deliberately focused on rare disease specific guidances and excluded any general guidance that is applicable to any product development, this includes ICH guidances.

Approval of new medicines is one key factor for the availability of new medicines, however, ensuring that medicines are both accessible and affordable is the other part of the equation. Around 263 to 446 million people worldwide live with a rare disease at any given time, many of these conditions are debilitating or life-threatening and about half affect children (2, 3). This highlights a strong need to provide patients with effective therapies. We therefore also considered the healthcare payer environment over the same period to see if the incentives to develop new medicines for rare diseases were mirrored by incentives for these therapies to be both accessible and affordable.

### Regulatory requirements: changes and trends

The introduction of the Orphan Drug Act in the USA in 1983, followed by adoption of the Orphan Drug Regulation No 141/2000 in the EU in 2000, changed the landscape of drug development for rare diseases (1, 4). Both the FDA and the EMA subsequently issued multiple guidances and programs to help drug developers navigate implementation of respective Act and Regulation and provided various incentives to encourage drug development for rare diseases. To evaluate the potential impact that guidances could make on the development of treatments for rare diseases we conducted a search of EMA and FDA websites and identified rare disease specific guidances. 35 FDA guidances and 12 EMA guidances were identified, all guidances are listed under Reference section for ease of the review (5–52). In our opinion, the number of published guidances demonstrates interest and support for the rare disease community by both the EMA and FDA. However, the fact that FDA published almost 3 times more guidances than EMA may indicate that FDA has more dedicated resources and this could encourage sponsor to prioritize engagement with FDA during the product development, approval and access strategy. For ease of comparison and to avoid duplication we excluded general guidances that are applicable to product development for all products. For example, ICH guidances on quality, efficacy and safety are deliberately excluded from the evaluation, as all sponsors should consider ICH guidances during drug development, regardless of whether the drug is being developed for treatment of rare disease or not. It should be noted that while the FDA has a website on Guidance Documents for Rare Disease Drug Development, the EMA guidelines relevant to rare diseases are published separately and could be found through a search of general guidances on the EMA website (53, 54).

**Figure 1:**
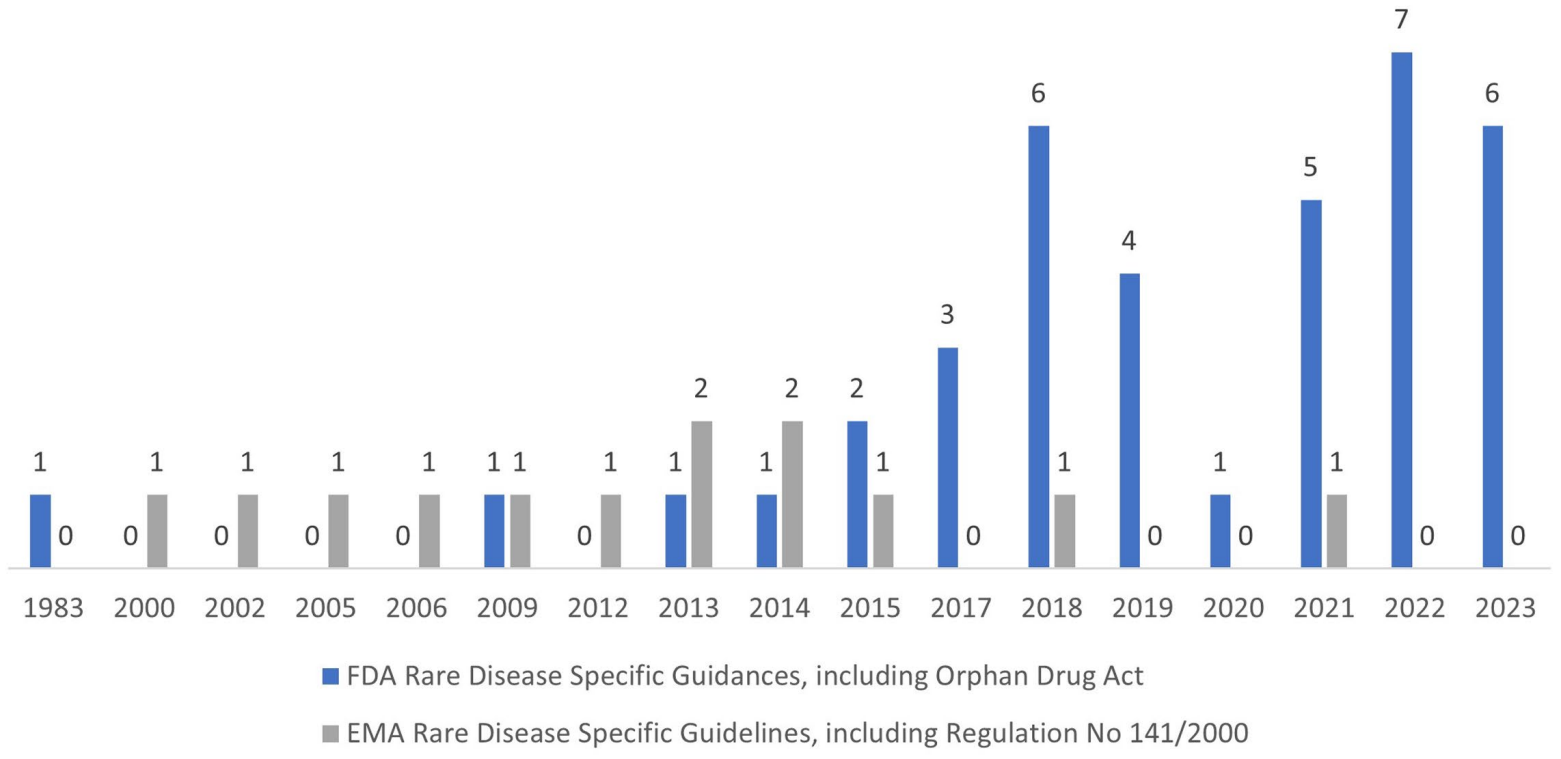
FDA and EMA rare disease specific guidances, including Orphan Drug Act and Regulation No 141/2000.

The summary of guidances and the trend in guidance publication are presented in Figure 1 with the detailed titles and dates of publications presented in the Reference section (1, 4–53). There was a notable uptick in the annual number of guidances published by the FDA starting from 2015, albeit with an obvious drop in 2020 when everyone was focused on addressing the COVID-19 pandemic. Rare disease guidances publication from the EMA has been consistent since the 2000. In our opinion the most impactful guidances are the ones that encourage sponsor to use innovative and collaborative approaches to drug development, for example, FDA Draft Guidance Pediatric Rare Diseases-A Collaborative Approach for Drug Development Using Gaucher Disease as a Model (12) and EMA Guideline on clinical trials in small populations (43). The evaluation of regulatory guidances reveals some differences in FDA and EMA approaches to providing regulatory directions. In general, the FDA guidances focus on common issues and specifics of product development for all rare diseases with the exception of disease specific guidances for Duchenne and Gaucher diseases, the latter of which was done in collaboration with the EMA. The EMA issues more disease specific guidances, with the intent to help sponsors with drug development guidance for specific diseases. For the purpose of this paper, we only focused on guidances, however, it should be noted that both Agencies expanded their work outside of just publishing guidances. There were multiple incentives and engagements with major stakeholders in rare disease drug development in addition to collaborative efforts between EMA and FDA in a Rare Diseases Cluster, which was established in 2016. Another noticeable EU initiative, supported by EMA, is Medicines Adaptive Pathways to Patients (MAPPs) which is a concept that seeks to foster access to novel/beneficial treatments for the right patient groups at the earliest appropriate time in the product life-span, in a sustainable fashion. MAPPs is not an official designation and is not intended to create new regulatory or legal frameworks (55). Both FDA and EMA have multiple incentives to facilitate drug development for life-threatening and debilitating diseases. Although, the discussion of these incentives is outside of the scope of this paper, a high-level overview of these incentives and timeline of implementation is presented in Table 1. These incentives in combination with regulatory guidances created a supportive network for rare diseases drug development and reflected in significant increase in orphan drug designation and approvals. For example, FDA approved 470 orphan drugs in the period 2013–2022, which is a 6 fold increase compared to the 80 orphan drugs approvals in the period 1983–1992 (56).

**Table 1:** Summary of FDA and EMA incentives to facilitate drug development for rare diseases.

Description	Incentives	Year of implementation
EMA		
Orphan drug designation	Protocol assistance Access to the centralized authorisation procedure Ten years of market exclusivity Additional incentives for small and medium-sized enterprises (SMEs) Fee reduction Grants Incentives in member states	1999 under Orphan Drug Regulation (4, 57),
PRIME	Enhanced support from EMA, tailored to the relevant stages of development Confirmation of potential accelerated assessment	2016 as European commission initiative (94)
Advanced therapy medicinal products (ATMP)	Enhance scientific support (PRIME) for ATPs Facilitate approval of clinical trials Specific action plan for SMEs Foster increased interaction between EMA and EUnetHTA on ATMPs 65% fee reduction for a request for scientific advice for ATMPs (90% for SMEs); 90% fee reduction for the certification procedure.	2008 under EC Regulation No 1394/2007 (95, 104)
Support for micro, small and medium-sized enterprises (SMEs)/SME office	Direct contact the SME office for questions about regulations, administrative requirements or procedures Request a briefing meeting Receive translation assistance for the product information into all official EU languages Receive guidance on clinical data publication; Stay up to date with SME newsletters; Participate in training events; Receive support with looking for academic partners in the paediatric-medicine field	2005, Commission Regulation (EC) No 2049/2005 (105)
Conditional marketing authorization (CMA)	Fast-track approval of a medicine that fulfils an unmet medical need Must fulfil specific obligations within defined timelines	2004, Regulation (EC) No 726/2004, further elaborated in Regulation (EC) No 507/2006 (106).
Exceptional circumstances	Marketing authorization granted to medicines where the applicant is unable to provide comprehensive data on the efficacy and safety under normal conditions of use, because the condition to be treated is rare or because collection of full information is not possible or is unethical	2004, Article 14 (8) of the Regulation (EC) No 726/2004 (107)
Accelerated assessment (AA)	Reduce the timeframe to 150 days if the applicant provides sufficient justification for an accelerated assessment	2004, Recital 33 and Article 14 (9) of Regulation (EC) No 726/2004 (108)
Parallel EMA/FDA scientific advice (PSA)	Receiving feedback from both agencies and ability to align product development with both EMA and FDA expectations	2021, collaborative initiative between EMA and FDA (109)
Parallel consultations EMA/HTA (EUnetHTA)	Streamlined procedure for applicants; Increased mutual understanding and problem-solving ability between EMA and HTA bodies through a more structured interaction; Improved coordination with, and greater participation of HTA bodies in parallel consultations through EUnetHTA 21’s committee for scientific quality and consistency in its configuration for joint scientific consultations (CSCQ JSC)	2022, collaborative initiative between EMA, HTAs and EUnetHTA (110)
FDA		
Orphan drug designation	More frequent communication with FDA Tax credits for qualified clinical testing Waiver of NDA/BLA user fees Eligibility for 7-year marketing exclusivity (“orphan exclusivity”) upon marketing approval	1983 under Orphan Drug Act (1)
Fast track designation	More frequent interactions with FDA Eligibility for accelerated approval and priority review Rolling review	1997 under Food and Drug Administration Modernization Act of 1997 (FDAMA) (111)
Breakthrough therapy designation	Eligible for all Fast Track designation features Intensive guidance on an efficient drug development program, beginning as early as Phase 1 Organizational commitment involving senior FDA managers	2012 under Food and Drug Administration Safety and Innovation Act (FDASIA) (112)
Regenerative medicine advanced therapy designation (RMAT)	Eligible for all the benefits of the fast track and breakthrough therapy designation programs	2016 under 21st Century Cures Act (113, 120)
Priority review	Shorter clock for review of marketing application 6 months compared to 10 months	1992, under the Prescription Drug User Act (PDUFA) (114)
Accelerated approval	Approval based on an effect on a surrogate endpoint or an intermediate clinical endpoint that is reasonably likely to predict a drug’s clinical benefit	2012 under Food and Drug Administration Safety and Innovation Act (FDASIA) (115)
Real-time oncology review (RTOR)	Expedite drug approval review: FDA reviews clinical data throughout the development process, and before a company formally applies for approval	2018 under collaboration of FDA Oncology Center of Excellence (OCE), with the Office of Oncologic Diseases (OOD) (116, 119)

In totality the cumulative efforts that were made by both EMA and FDA resulted in significant increase in drugs for rare diseases, as presented in the next section.

### Medicines for rare diseases: overview

**Figure 2:**
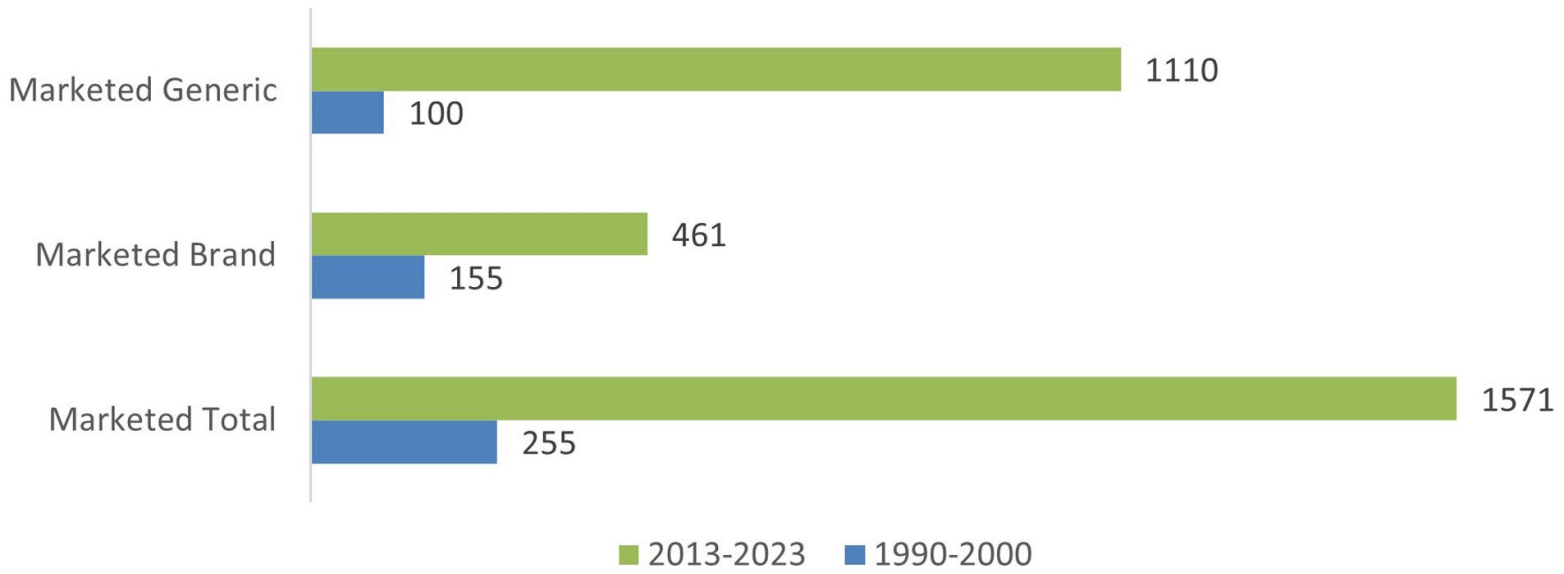
Comparison of rare disease products in USA between 1990–2000 and 2013–2023 by type of product: brand or generic.

**Figure 3:**
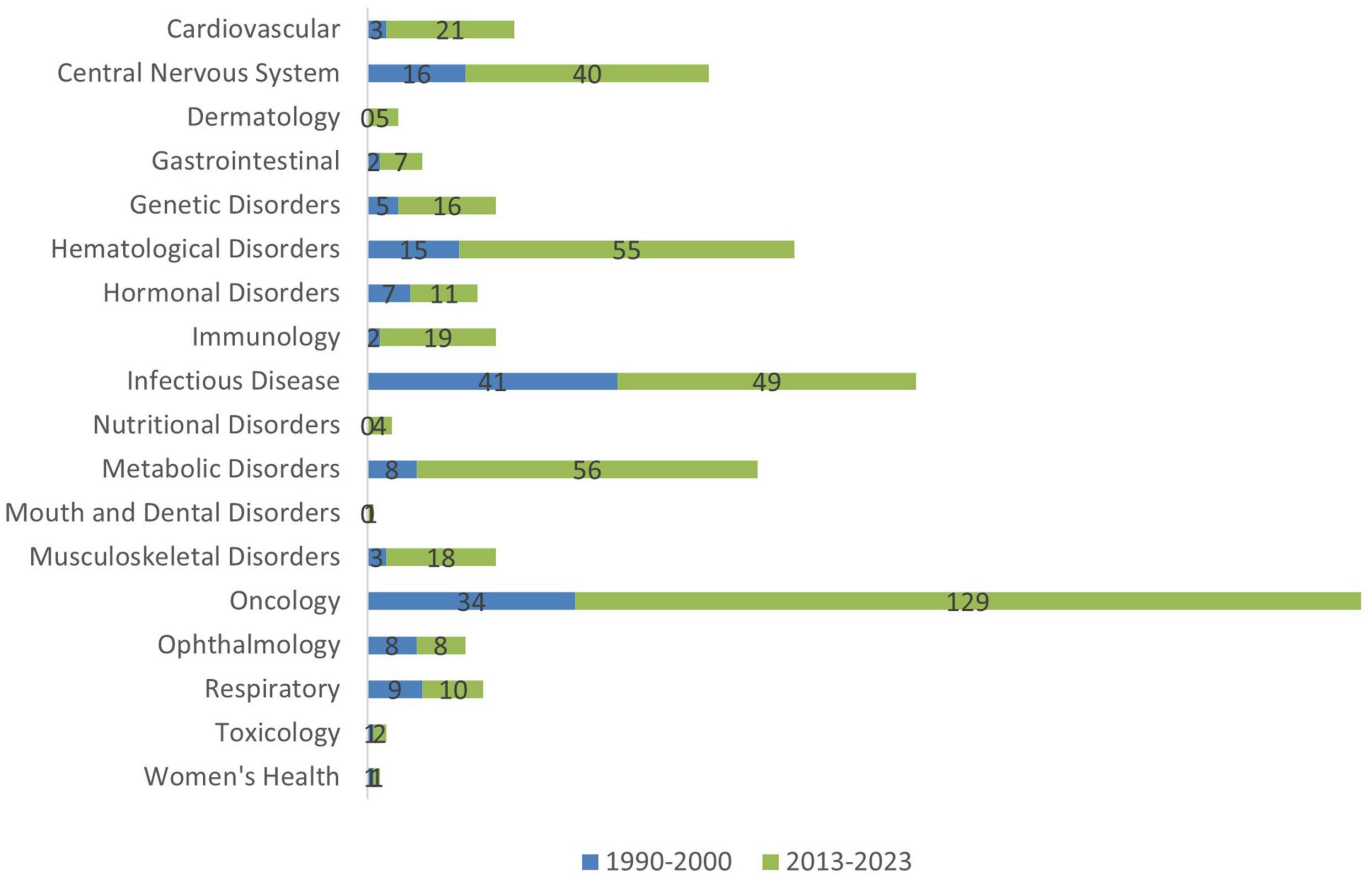
Comparison of marketed brand products for rare disease products by therapeutic area in USA.

To evaluate the impact of changes in the regulatory and access landscapes on the number of rare disease treatments available in US and EU we accessed the GlobalData system. As a “baseline” we extracted all marketed products for the treatment of rare diseases that were listed in the database from 1990–2000, before the ODA and Orphan Drug Regulations in USA and EU, respectively could have reasonably impacted drug approvals. To evaluate the impact of changes in regulatory and access environment we used the same criteria and extracted products for rare disease treatments marketed from 2013–2023. The 1990–2000, timeframe was selected because the drugs marketed during this period were unlikely to have benefited from orphan drug legislative incentives. The 2013–2023 timeframe was chosen, because drugs marketed during this period were considered to have both the time and opportunity to have benefited from orphan drug incentives. We selected all types of products for treatment of rare diseases, including but not limited to small molecules, biologics and combination products. During the EU data analysis it was not possible to establish a clean dataset for EU marketed products due to multiple factors, including but not limited to placement of the same product on the market under duplicate licenses. Therefore, the analysis proceeded with the data from the US only, however, some specific examples of access considerations in EU were evaluated and presented further in this publication. The graphical representation is provided in Figures 2, 3. Figure 2 depicts the total number of products marketed in the USA during the two periods as well as a breakdown of the data by branded and generic products.

Overall, the number of marketed products per decade increased from 255 in 1990–2000 to 1,571 in 2013–2023, a 6-fold increase. This increase clearly indicates that incentives and support, provided to rare disease drug developers, including, but not limited to increase in guidances, had a significant impact on access. When looking at the changes for branded innovator products and generic products separately, both showed a marked increase in availability (3-fold and 11-fold respectively) between 1990–2000 and 2013–2023. The increase in the availability of new treatments is much higher in some therapeutic areas than others, as presented in Figure 3. Therapeutic areas showing a higher increase in new products for Rare Diseases included oncology, metabolic, hematological disorders and central nervous system and cardiovascular disorders. These areas are also ones which in general are current areas of focus for pharmaceutical R&D. Despite the clear increase in the number of products reaching the market for Rare Diseases in 2013–2023 access to these products has presented a variety of challenges, as discussed in the next section.

### Access challenges

Timely access to medicines is essential to reduce morbidity and mortality of orphan diseases. However, regulatory approval still does not guarantee access for patients. According to the European Federation of Pharmaceutical industries and Associations there is still considerable variation in time across the EU Member states between the authorization and reimbursement of new medicines with mean time to reimbursement ranging from 102 days in Germany to 993 days in Poland (57).

The US Orphan Drug Act, and the European Orphan Medicinal Product Regulation were big steps toward greater availability of orphan medicines. While orphan designations directly translate into easier access to therapies via compassionate use, and early access programs, the effective access to orphan medicines in a targeted population of interest remains complex as healthcare decision makers need to allocate resources for drug funding within already constrained healthcare budgets. The complexity of access challenges is multifaceted, and may include:

- Requirements for robust evidence by HTA Assessors; Due to low patient numbers, nature of the condition, absence of standard of care orphan disease randomized controlled trials (RCTs) have inherent limitations which may hinder demonstrating therapeutic value for a new therapy, e.g., small sample size, short study duration, use of biomarkers or surrogate study end-points, lack of appropriate comparator in the control arm; [recent examples include elafibranor for the treatment of primary biliary cholangitis (regulatory approval in 2024), talquetamab for the treatment of relapsed refractory multiple myeloma after four prior lines of therapy (regulatory approval in 2023)] which despite regulatory approval failed to demonstrate additional clinical benefit during French HTA assessment [ASMR *Amélioration du service médical rendu* V (absent)] (58–63).
- High cost of therapy, resulting in challenges to demonstrate economic value to local decision-makers (e.g., impact to local healthcare budget high, cost-effectiveness above locally acceptable willingness-to-pay threshold); (the highest costs among orphan drugs are often attached to gene therapies for examples etranacogene dezaparvovec for severe and moderately severe hemophilia B or exagamglogene autotemcel for the treatment of *β*-thalassemia and sickle cell disease with price tag of $3.5 M and $2.2 M per single administration) (64–69).
- Assessors knowledge and capacity; limited capacity, and/or limited clinical or technical expertise to assess advanced statistical and health economic methods submitted within product evidence package
- Legislation and policy; lack of uniform value assessment and appraisal process across markets, lack of innovative access models to manage “one-time-administration” potentially curative advanced therapy medical products

Although it is beyond the scope of this publication to describe the evolution of the pricing, reimbursement and access landscape of both the USA and the EU member countries, we have explored access context of the leading European markets such as Germany and France to compare to the evolution of regulatory policies previously described. Although reimbursement legislation and policies vary across the EU member states, they all provide public healthcare coverage. We delve into Germany and France as the first two countries in terms of pharmaceutical market value in Europe (€47.588 billion and €32.077 billion sales in 2021, respectively) (70). Among the key five European markets (4 EU member states and UK) Germany and France had historically the best access indicators for orphan medicines (number of medicines reimbursed and months to reimbursement) (71). In both countries reimbursement is linked to the outcomes of national health technology assessment (HTA) and medicines with positive HTA recommendation are funded through healthcare payer budgets. In contrast to primarily cost-effectiveness HTA framework, both countries have a system driven by assessment of clinical benefit and reimbursed price based on the demonstrated level of additional clinical benefit over standard of care therapies, German policies incentivize access to all medicines through a 6-month free pricing mechanism and availability immediately upon EMA regulatory approval (117). Moreover, access to orphan drugs is facilitated given that these medicines are exempt from the full HTA (i.e., the need to demonstrate benefit versus an appropriate comparator) and approval is granted based on a minimum level of additional benefit. Orphan medicines are required to undergo full HTA only after exceeding the threshold of €30.0 million annual sales (117). In contrast, there is no designated market access pathway for orphan medicines in France, but there are early access (EA) mechanisms in place allowing innovative medicines to be funded prior to the EMA regulatory approval and/or prior to the completion of the HTA (72). The EA mechanism has proven particularly effective for rare genetic conditions that are highly debilitating, especially those with early-onset: since 2016 the EA program facilitated access of three innovative therapies for spinal muscular atrophy (SMA) type 1, 2 or 3: 48 patients were enrolled in the nusinersen EA program (Oct 2016–Jun 2017), 14 patients enrolled in onasemnogene abeparvovec EA program of (Jun 2019–May 2020) and 110 patients enrolled in risdiplam EA program (Dec 2020–Apr 2021) (73, 74).

While there are additional similarities and differences in the reimbursement processes between Germany, France, and other EU member states, these are not expected to significantly impact access to orphan medicines and are outside the focus of this publication.

In the US, access to orphan drugs, like other drug products, is governed by the major purchasers of healthcare in the US which are largely the government programs of Medicare, Medicaid, and health insurance exchanges, and the employer-sponsored insurance market. Typically, orphan drugs require prior authorization, which is a mechanism payers use to manage utilization and ensure that physician drug choices are clinically appropriate and within label. Orphan drugs, due to their high cost, usually have fairly detailed prior authorizations, which may require submission of clinical documentation and justification of medical necessity. Prior authorizations for orphan drug products often include some key inclusion/exclusion criteria from clinical trials, in an effort to achieve the clinical outcomes seen in that setting. While a critical tool to ensure appropriate use, prior authorizations can result in delays in treatment. Most patients in the US market experience some form of cost sharing for drugs as well, which can create another barrier to access.

In Germany and France, the reimbursement of orphan medicines in therapeutic areas in which an increase in availability was noted (oncology, central nervous system, hematological and metabolic disorders) has largely followed the regulatory pace with some delays in time to effective access driven by the time taken for the HTA assessment and price negotiation (e.g., in France). The areas with more prominent differences between the number of regulatory approvals, access in the US and that in the EU members states are rare genetic diseases with recent approvals of novel gene therapies. Among the 7 non-oncology gene therapies which had FDA and EMA regulatory approvals (<etranacogene dezaparvovec for severe haemophilia B, betibeglogene autotemcel for transfusion-dependent beta-thalassemia, onasemnogene abeparvovec for spinal muscular atrophy, valoctocogene roxaparvovec for severe hemophillia A, voretigene neparvovec for retinal dystrophy, exagamglogene autotemcel for sickle cell disease and transfusion-dependent *β*-thalassemia, and lovotibeglogene autotemcel for metachromatic leukodystrophy), all 7 are funded and available in the US, 5 received positive HTA recommendations in Germany and France (etranacogene dezaparvovec, onasemnogene abeparvovec, valoctocogene roxaparvovec, atidarsagene autotemcel, and voretigene neparvovec), but information on effective access including price were not identified for etranacogene dezaparvovec, nor valoctocogene roxaparvovec in France (64–67, 73, 75–91, 118). Potential uncertainties associated with perceived drug value and long-term treatment benefits were mitigated with mandatory data collection and/or re-assessment upon more evidence being available. In addition to the evidence driven hurdles during the HTA process, access to orphan medicines in the EU-member states, such as Germany and France, may be driven by manufacturer decision to opt out in instances when healthcare payer acceptable price is not commercially viable for manufacturers. Although these are rather exceptions, after unsuccessful price and reimbursement negotiations in Germany Bluebird decided to focus betibeglogene autotemcel efforts on the US market citing “*challenges of achieving appropriate value recognition and market access in Europe*” (92). Which lead to complete withdrawal of the EMA authorization in 2022 (75).

Manufacturers of orphan drug products sold in the US are free to set and change price according to market demand, and all payers are compelled to provide access to products deemed medically necessary (or justify why it is not medically necessary for that patient). This has resulted in multi-million dollar prices for one-time administrations of gene therapies, albeit with substantial authorization criteria from payers, both government and private. Yet, these are sometimes life-saving and life-changing therapies, so from a health economic perspective, many gene therapies are cost-effective in the short and long term views.

The pricing of many orphan drug products in the US has created an access environment in which they are likely to be covered for eligible patient populations, but with real financial impact to public and private payers. This has spurred the development of risk-sharing agreements in which failure to achieve a clinical outcome is tied to some level of financial remuneration. These agreements, when in place, offer some downside protection to payers while also ensuring access to these medications for patients.

Overall, access to orphan medicines is complex and multi-faceted. Local healthcare decision makers have made steps and progress in implementing mechanisms to facilitate access, but the work is still ongoing in making therapies timely available to all the patients in need.

## Discussion

It is clear from the data examined that the implementation of legal frameworks and incentives, the availability of guidance documents and the partnerships and support provided by global regulatory agencies has created an environment which has clearly resulted in an increase in the regulatory approval of new medicines for rare diseases, although the speed of this change was slow, despite of the positive trend, for example there were 6 folds increase in rare disease drug approvals by FDA in the period from 2013 to 2022 in comparison to 1983–1992 (56).

In the US and Europe, regulatory agencies support programs (e.g., EMA’s support for early access and four FDA’s Expedited Programs: Fast Track Designation, Breakthrough Therapy Designation, Accelerated Approval and Priority Review Designation) are available to facilitate and expedite clinical development and regulatory approval with the aim to foster timely access to patients with serious conditions and clear unmet medical need (93, 94). The programs consider iterative processes including early dialog with manufacturers in preparation of technical and scientific aspects of the regulatory submissions (93, 94).

In the US over the last two decades, both public and private payers have dealt with the advent of high-cost, clinically innovative orphan drug products by attempting to manage access as close to the clinical trial population as possible. While sometimes onerous and resulting in delays in therapy, this approach has worked well to ensure that appropriate patients are receiving medically necessary treatments.

More recently, with the launch of gene therapies in orphan disease areas, risk sharing agreements have become more commonplace to address the financial impacts on payers (95). While in its infancy, this represents movement toward the objective of aligning payment for value, as defined by clinical outcomes.

In Europe, local decision makers have introduced policies and mechanisms to facilitate access to medicines while managing constrained budgets, yet there are still hurdles to overcome. In 2013, the Mechanism of Coordinated Access to Orphan Medicinal Products (MoCA) was established at the European Level between volunteering EU stakeholders and developers of Orphan Medicinal Products with the aim to support the exchange of information, enable informed decisions on pricing and reimbursement at EU-member state level and assess the value of orphan medicines based on a transparent framework (96, 97). MoCA created a voluntary and flexible framework for non-binding dialog between different stakeholders with the main objective to *“support more equitable access to authorized therapies for people living with rare diseases, rational prices for payers and more predictable market conditions for Orphan Medicinal Products developers”* (98). During the 10-year MoCA pilot program 23 orphan products were discussed involving industry, payer/HTA and patient representatives. Although informal and non-binding, one of the key drivers of accelerating access to orphan medicines of this pilot is the collaboration between industry, payers, and patient advocacy groups with a common goal to ensure that clinical development addresses the unmet needs and ensures access once approved. In 2022, the European Federation of Pharmaceutical Industries and Associations (EFPIA) and the European Organization for rare Diseases (EURORDIS) issued a joint statement bringing forward proposals to bolster HTA process and pricing and reimbursement framework for orphan drugs (99). Finally, the new Joint Clinical Assessment Process (starting in 2025 and applicable to orphan medicines from 2028 on) aims to ensure a uniform clinical assessment at the EU level and facilitate HTA collaboration across EU member states with the final goal of accelerating access to medicines (98, 100).

These changes reflect a growing recognition of the importance of addressing the needs of rare disease patients and a concerted effort to balance innovation with affordability and access.

In looking at the more than three decades over which progress on the number of new medicines for rare diseases has been made, it is difficult to conclude that the implementation of legal regulations alone was sufficient to drive change. It was only when the regulatory agencies, pharmaceutical industry, patient groups and other stakeholders worked together that progress became significant. The requirements around the need to ensure safety, efficacy and quality for orphan drugs is not reduced because patient numbers are small. However, an alignment was created on acceptable, innovative ways to meet these requirements in the context small patient numbers, through the use of non-traditional data sources and integrative evidence approaches. This enables sponsors to develop new orphan drugs in collaboration with regulators and patients and helps to ensure that the needs of both are met.

In contrast to the partnership among multiple stakeholders and the increasing progress of new drug development paradigms in the development and regulatory approval of orphan drugs, the subsequent access to those medicines over the same period was still globally fragmented. In Europe the EFPIA and EURORDIS proposal and the new Joint Clinical Assessment Process should increase the uniformity of clinical assessment and provide a pricing and reimbursement framework for orphan drugs. These changes reflect a growing recognition of the importance of addressing the needs of rare disease patients and a concerted effort to balance innovation with affordability and access. It is hopeful that a similar collaborative approach, that was successful in the regulatory approval space, if successfully translated into the market access and pricing arena would result in a similar step change in the timely access to new medicines for rare diseases. It may already be too late for companies with gene therapies for rare diseases. These companies struggle to make therapies profitable given a small pool of eligible patients and challenges in scalability. Following layoffs in 2024 and struggles with cash flow, Bluebird Bio, which has been a pioneer in gene therapy development, announced in February 2025 its acquisition by Carlyle to secure a financial path forward for the company (68). At the same time Pfizer announced its decision to stop the commercialization of Beqvez (101). This news follows previous indications that other companies are also struggling with multiple companies pulling their development programs in this space, CSL reporting slower-than-expected sales for Hemgenix and BioMarin’s decision to focus commercialization of Roctavian on markets where it is reimbursed (102, 103). We hope that these examples of the challenges to successful commercialization will bring more public and government attention in order to encourage establishment of innovative approaches to the ensure commercial success for gene therapies.
